# Na/K-ATPase as a target for anticancer drugs: studies with perillyl alcohol

**DOI:** 10.1186/s12943-015-0374-5

**Published:** 2015-05-15

**Authors:** Diogo Gomes Garcia, Hugo Caire de Castro-Faria-Neto, Camila Ignácio da Silva, Kauê Francisco Correa de Souza e Souza, Cassiano Felippe Gonçalves-de-Albuquerque, Adriana Ribeiro Silva, Lidia Maria da Fonte de Amorim, Aline Soares Freire, Ricardo Erthal Santelli, Luan Pereira Diniz, Flávia Carvalho Alcantara Gomes, Mauro Velho de Castro Faria, Patrícia Burth

**Affiliations:** Laboratório de Imunofarmacologia, Instituto Oswaldo Cruz, Fundação Oswaldo Cruz, Rio de Janeiro, RJ Brazil; Departamento de Biologia Celular e Molecular, Instituto de Biologia, Universidade Federal Fluminense, Niterói, RJ Brazil; Departamento de Química Analítica, Instituto de Química, Universidade Federal do Rio de Janeiro, Rio de Janeiro, RJ Brazil; Instituto de Ciências Biomédicas, Universidade Federal do Rio de Janeiro, Rio de Janeiro, RJ Brazil; Departamento de Medicina Interna, Faculdade de Ciências Médicas, Universidade do Estado do Rio de Janeiro, Rio de Janeiro, Brazil

**Keywords:** Na/K-ATPase, Perillyl alcohol, JNK, U87 and U251 glioma cells

## Abstract

**Background:**

Na/K-ATPase (NKA) is inhibited by perillyl alcohol (POH), a monoterpene used in the treatment of tumors, including brain tumors. The NKA α_1_ subunit is known to be superexpressed in glioblastoma cells (GBM). This isoform is embedded in caveolar structures and is probably responsible for the signaling properties of NKA during apoptosis. In this work, we showed that POH acts in signaling cascades associated with NKA that control cell proliferation and/or cellular death.

**Methods:**

NKA activity was measured by the amount of non-radioactive Rb^+^ incorporation into cultured GBM cell lines (U87 and U251) and non-tumor cells (mouse astrocytes and VERO cells). Cell viability was measured by lactate dehydrogenase levels in the supernatants of POH-treated cells. Activated c-Jun N-terminal Kinase (JNK) and p38 were assessed by western blotting. Apoptosis was detected by flow cytometry and immunocytochemistry, and the release of interleukins was measured by ELISA.

**Results:**

All four cell types tested showed a similar sensitivity for POH. Perillic acid (PA), the main metabolite of POH, did not show any effect on these cells. Though the cell viability decreased in a dose-dependent manner when cells were treated with POH, the maximum cytotoxic effect of PA obtained was 30% at 4 mM. 1.5 mM POH activated p38 in U87 cells and JNK in both U87 and U251 cells as well as mouse astrocytes. Dasatinib (an inhibitor of the Src kinase family) and methyl β-cyclodextrin (which promotes cholesterol depletion in cell membranes) reduced the POH-induced activation of JNK1/2 in U87 cells, indicating that the NKA-Src complex participates in this mechanism. Inhibition of JNK1/2 by the JNK inhibitor V reduced the apoptosis of GBM cells that resulted from POH administration, indicating the involvement of JNK1/2 in programmed cell death. 1.5 mM POH increased the production of interleukin IL-8 in the U251 cell supernatant, which may indicate a possible strategy by which cells avoid the cytotoxic effects of POH.

**Conclusions:**

A signaling mechanism mediated by NKA may have an important role in the anti-tumor action of POH in GBM cells.

**Electronic supplementary material:**

The online version of this article (doi:10.1186/s12943-015-0374-5) contains supplementary material, which is available to authorized users.

## Background

Na/K-ATPase (NKA) is a membrane-associated protein complex found in animal cells that couples the energy stored in ATP molecules to the transport of Na^+^ and K^+^ across cell membranes. This transport produces an electrochemical gradient that is essential for maintaining the cell membrane potential and the excitable activities of muscle and nerve cells [1-3]. Apart from its function as an ion pump, NKA is also a signal transducer. This enzyme interacts with different signaling proteins in caveolae, including Src tyrosine kinase, PKC, PKA, PI3K, caveolins and EGFR [4-8]. This set of proteins associated with NKA is called the signaling complex or signalosome, which is restricted to caveolae [5,7-12].

The activation of the NKA-Src complex is the starting point for signaling after the binding of cardiac glycosides to NKA. Ouabain binding regulates the interaction between NKA and caveolin, which stimulates the cytoplasmic Src kinase. Activated Src transactivates EGFR, which recruits adapter proteins, leading to further activation of the Ras-Raf-MAPK cascade [9,12]. The three major members of the mitogen-activated protein kinase family (MAPKs) are extracellular signal-regulated kinase (Erk), c-jun N-terminal kinase (JNK) and p38. In general, activated Erk controls cell proliferation and differentiation. On the other hand, JNK and p38 signaling promotes cell proliferation, invasion, survival, migration, growth arrest and apoptosis [13]. However, the intracellular signaling triggered by cardiac glycosides depends on the cell type, exposure time and drug concentration [14,15].

The effects of cardiac glycosides on the activity of NKA and the use of these glycosides in the therapy of cardiovascular diseases have been broadly publicized. However, some studies have shown low mortality rates in cancer patients treated with cardiac glycosides, particularly in women with breast cancer [16]. The fact that NKA also acts as a signal transducer has sparked new interest in the properties of cardiac glycosides as anticancer drugs [17]. Moreover, NKA activity is already changed during the early stages of tumorigenesis, even before morphological evidence can demonstrate the presence of tumors [18,19]. Some studies have suggested that the suppression or stimulation of NKA isoforms, mainly α1 and α3, depends on the type of cancer in question. For example, the expression of NKA α1 is stimulated in cancers of the brain, lung and skin or inhibited in bowel and bladder cancers. On the other hand, NKA α3 is mainly stimulated in colon and rectal cancers [20,21]. Several *in vivo* and *in vitro* studies have confirmed these observations, and substances based on the structures of cardiac glycosides have already been used in clinical trials for cancer treatment [22-24]. These findings suggest that new anticancer agents that act on NKA can be developed, as this enzyme may be an important target for anticancer therapy [25]. This is especially the case for the α1 subunit of NKA in apoptosis-resistant glioblastoma cells [26,27].

The importance of NKA in anticancer therapy has also been suggested using compounds unrelated to the cardiac glycoside structure, such as the monoterpene perillyl alcohol (POH) [28,29]. POH is found in essential oils from various plants that have chemopreventive and chemotherapeutic activities against different tumors, including glioblastomas (GBM), the most common and malignant human brain tumor [30-33]. GBM is characterized as a high-grade astrocytoma (grade IV) that presents an infiltrating ability and the absence of limitation. Our previous studies conducted in both membrane preparations and in glioblastoma cells have shown that the POH is an NKA inhibitor with higher specificity for the α1 subunit than the predominant brain isoforms (α2 and α3) [28]. This fact may be interesting because this isoform was described in the literature as a mediator of signal transduction mechanisms [26]. Due to the involvement of NKA in numerous cellular functions, changes in the activity and expression of this enzyme may be related to the pathogenesis of many diseases, making this enzyme a powerful therapeutic target. Therefore, our aim was to determine whether POH might act on signaling cascades modulated by NKA, thus controlling cell proliferation and/or death.

## Materials and Methods

### Cell culture conditions

Astrocyte primary cultures were prepared from newborn Swiss mice following the procedure previously described by Gomes *et al*. (1999) [34]. U87 and U251 cell lines (from human GBM) and VERO cell lines (from African green monkey kidneys) were cultured with DEMEM or DMEM-F12 (Invitrogen) supplemented with 10% heat-inactivated fetal bovine serum (Invitrogen) and antibiotics (100 U/mL penicillin, 100 U/mL streptomycin-Invitrogen) at 37°C in a humid atmosphere containing 5% CO_2_. The cells were grown to semi-confluence in plates containing 6 or 24 wells.

### Na/K-ATPase assay in intact cells

The NKA assay in cell culture was based on the measurement of non-radioactive Rb^+^ incorporation according to Gill *et al*. (2004) [35]. After the samples were digested with nitric acid, Rb^+^ measurements were done in an inductively coupled plasma-optical emission spectrometer (Jobin–Yvon), as described previously [28]. Different concentrations of POH, 4 mM perillic acid (PA) and 0.5 mM ouabain were added to specific wells. These drugs were obtained from Sigma. POH and PA were dissolved in dimethyl-sulfoxide (DMSO) and used in the cell treatments at maximum concentrations of 0.1%. After 30 min incubation at 37°C, the cells were washed three times with PBS, and 0.6 mL per well of the cell lysis solution (0.15% SDS) was added to each well. The NKA activity was expressed as the difference between Rb^+^ incorporation in the absence or presence of 0.5 mM ouabain.

### Evaluation of cytotoxicity

We used the Cytotoxicity Detection Kit (Doles) to quantitate cell death. This assay involves a colorimetric quantification of cell death based on the measurement of the activity of lactate dehydrogenase (LDH) released into the supernatant of damaged cells. POH (0.5, 1, 1.5, 2, 2.5 and 4 mM), 4 mM PA and 0.5 mM ouabain were added to specific wells. After 30 minutes or 24 hours incubation at 37°C, the supernatant was collected and the LDH activity was measured by spectrophotometry at 510 nm. As a control for the maximum LDH release, cells were treated with 0.1% triton-X100 in DMEM medium for 30 min before running the assay. Some controls were performed to define a possible interference of DMSO. The cell viability in control samples (in the presence of DMSO 0.1%) was defined as 100% and the amount of viable cells in the treated samples was expressed as a percentage of those in the DMSO controls.

### JNK and p38 phosphorylation

After 30 min incubation at 37°C, the cells were lysed in buffer (11 mM Tris–HCl, 170 mM NaCl, Triton X-100 1%) containing protease and phosphatase inhibitor cocktail tablets (Roche). 6x sample buffer (containing 25 mM Tris, 40 mL Glycerol (85% v/v), 28 mM SDS, 20 mL β-mercaptoethanol and 120 mg Bromophenol blue) was added. The cell lysates were clarified by centrifugation and the supernatant was used for western blot analysis. The total protein concentrations were determined by the Bradford method [36].

The total protein content (50 μg) was loaded in each lane and separated using 10% SDS-PAGE electrophoresis. The bands were detected using chemiluminescence (Kit Amersham™ ECL™Prime Western Blotting Detection Reagent; GE) with an X-ray film (Hyperfilm ECL; GE) and quantified using the Image Master 2D Elite 4.01 (GE). The band densitometric measurements were normalized to the corresponding SAPK/JNK1/2 or p38 MAPK levels, with the control samples set to 1. Treatment values were then expressed relative to the control levels. The primary antibodies used were monoclonal anti-phospho-SAPK/JNK1/2 and anti-SAPK/JNK1/2 (1:1000; Cell Signaling; ordering numbers #4671S and #9258S, respectively) and anti-phospho-p38MAPK (1:500) and anti-p38MAPK (1:1000; Cell Signaling, ordering numbers #4511 L and #9212 L, respectively). The U87 cells were also pretreated for 30 minutes with 10 nM dasatinib (LC Laboratories) and 7.5 mM β-cyclodextrin (Sigma). After this pretreatment, the cells were treated with POH.

### Determination of cytokine levels

U87 and U251 cells were treated with POH (0.5 and 1.5 mM), ouabain (1 μM) or lipopolysaccharide (LPS - 5 μg/mL) from *Escherichia coli* (O127:B8 - Sigma) for 1, 6 and 24 hours. The supernatants were analyzed for interleukin production (IL-1β, IL-6 and IL-8) and tumor necrosis factor (TNFα) using the specific monoclonal antibodies of the Immunoassay kit (R&D Systems) according to the manufacturer’s protocols.

### Cell death assay

U87 and U251 cells were pretreated for 30 minutes with JNK inhibitor V [1,3-Benzothiazol-2-yl-(2-((2-(3-pyridinyl)ethyl)amino)-4-pyrimidinyl)acetonitrile; Calbiochem], an inhibitor of JNK1/2 activation, before treatment with 0.5 mM POH and 0.5 mM POH plus 0.5 μM JNK inhibitor V. After 24 hours of incubation, the cells were suspended in annexin and propidium iodide binding buffer as specified in the TACS Annexin V-FITC apoptosis detection kit (R&D Systems). The samples were analyzed using a BD Accuri C6 flow cytometer (BD Biosciences). The BD Accuri software was used to determine the Annexin V-positive apoptotic cells.

### Caspase-3 activation

U87 and U251 cells were treated for 24 hours with 0.1% DMSO or 0.5 μM JNK inhibitor V (control groups) and 0.5 mM POH or 0.5 mM POH plus 0.5 μM JNK inhibitor V. The cells that received JNK inhibitor V were pretreated with this inhibitor for 30 minutes before treatment. After 24 hours of incubation, the cells were fixed with 4% paraformaldehyde for 15 min. After this period, the cells were extensively washed in PBS (phosphate buffered saline) and unspecific sites were blocked with 3% bovine serum albumin (BSA), 5% normal goat serum (NGS) and 0.2% Triton X-100 (Vetec) diluted in PBS for 1 hour before immunoreactions with the following primary antibody: rabbit anti-cleaved caspase-3 (1:100, Cell Signaling). After 12 hours, the cells were thoroughly washed with PBS and incubated with secondary antibodies for 2 hours at room temperature. The secondary antibody was Alexa Fluor 488 (goat anti-rabbit IgG, Molecular Probes; 1:300). The nuclei were counterstained with DAPI (4’,6-diamidino-2-phenylindole, dilactate; Sigma). Glass coverslips were mounted on glass slides using Faramount mounting media (DakoCytomation). The stained cells were visualized using a fluorescent optical microscope Nikon TE3000. The number of cells was analyzed using the Image J software. At least 2000 cells were analyzed per experimental condition.

### Statistical analysis

Prism 5.0 software (GraphPadInc, CA, USA) was used for graphical presentation and statistical analysis. The statistical analyses included Student’s t-test and One-way ANOVA followed by Newman-Keuls test. The data are expressed as the means ± standard deviation of at least three independent experiments. Significance was determined at P < 0.05.

## Results

### POH and PA effect on NKA activity

**Figure 1:**
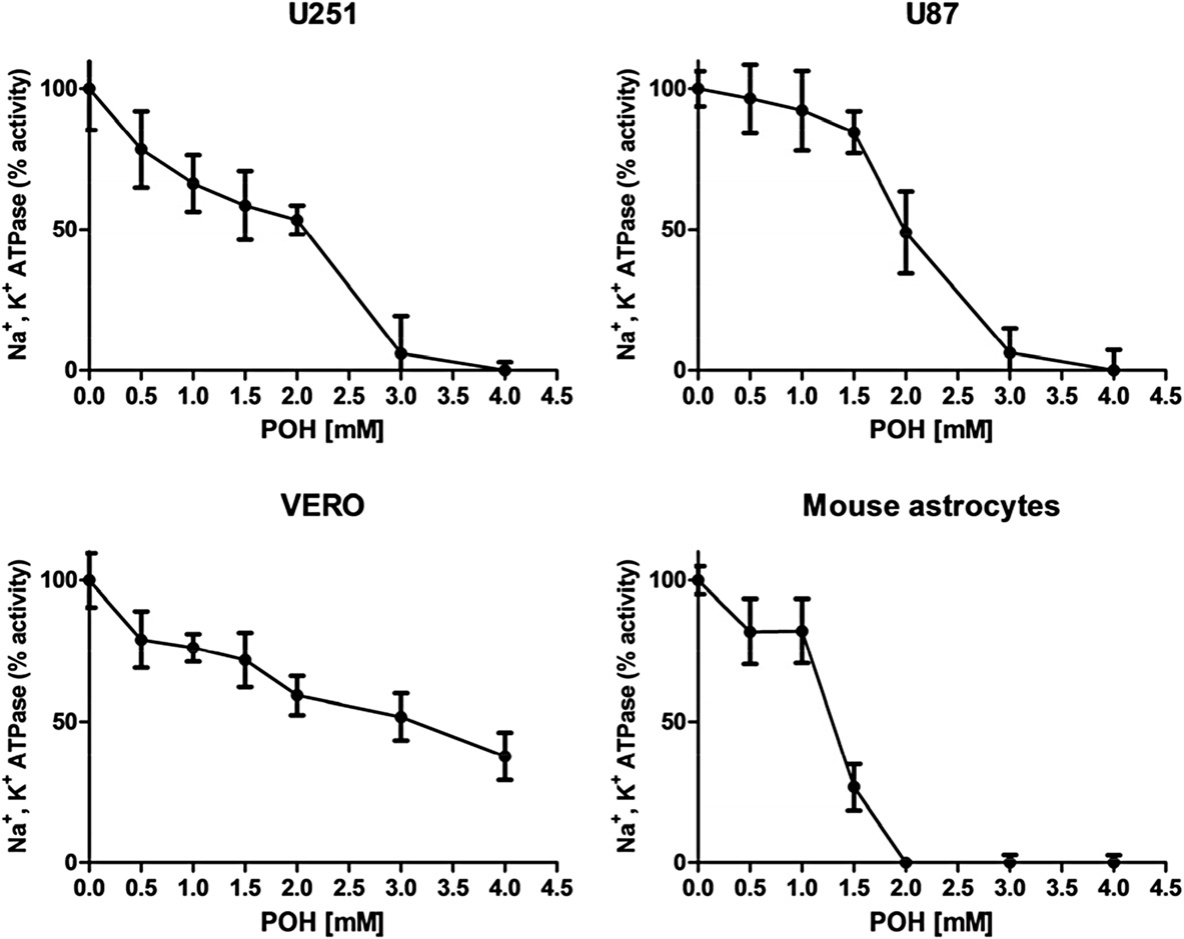
The effect of POH on the activity of NKA in the U251 and U87 cell lines, VERO cells and mouse astrocytes. Cells were treated with POH (0.5 - 4 mM) for 30 minutes. The NKA activity was expressed as the difference between the Rb^+^ uptake in the absence or presence of 0.5 mM OUA. Each point represents the means ± SD from at least four different experiments conducted in triplicate.

NKA activity in tumor and non-tumor cells was based on the incorporation of Rb^+^ by cells in the absence and presence of ouabain (OUA) 0.5 mM. NKA activity, which was expressed as the difference between Rb^+^ incorporation in the absence or presence of 0.5 mM OUA, was inhibited by POH (0.5, 1, 1.5, 2, 3 and 4 mM) in a dose-dependent manner (Figure 1). The IC_50_ values in the U251 and U87 cell lines were 1.8 and 2 mM, respectively. For the non-tumor cell lines VERO and mouse astrocytes, the IC_50_ values were 2.4 mM and 1.4 mM, respectively. Perillic acid (PA) showed no effect on NKA activity in all cell lines studied (Additional file 1).

### Cytotoxic effects of POH, PA and OUA after 30 min incubation

Cells (U251, U87, VERO and mouse astrocytes) were incubated for 30 minutes in the presence of POH, PA and OUA. Cell death was determined by measuring the LDH released in supernatant of treated and untreated cell cultures. POH decreased significantly the cell viability only at 4 mM. Likewise, 0.5 mM OUA and 4 mM PA did not affect the cell viability after a 30 min incubation period (Additional file 2).

### Cytotoxic effects of POH and PA after 24 hours incubation

**Figure 2:**
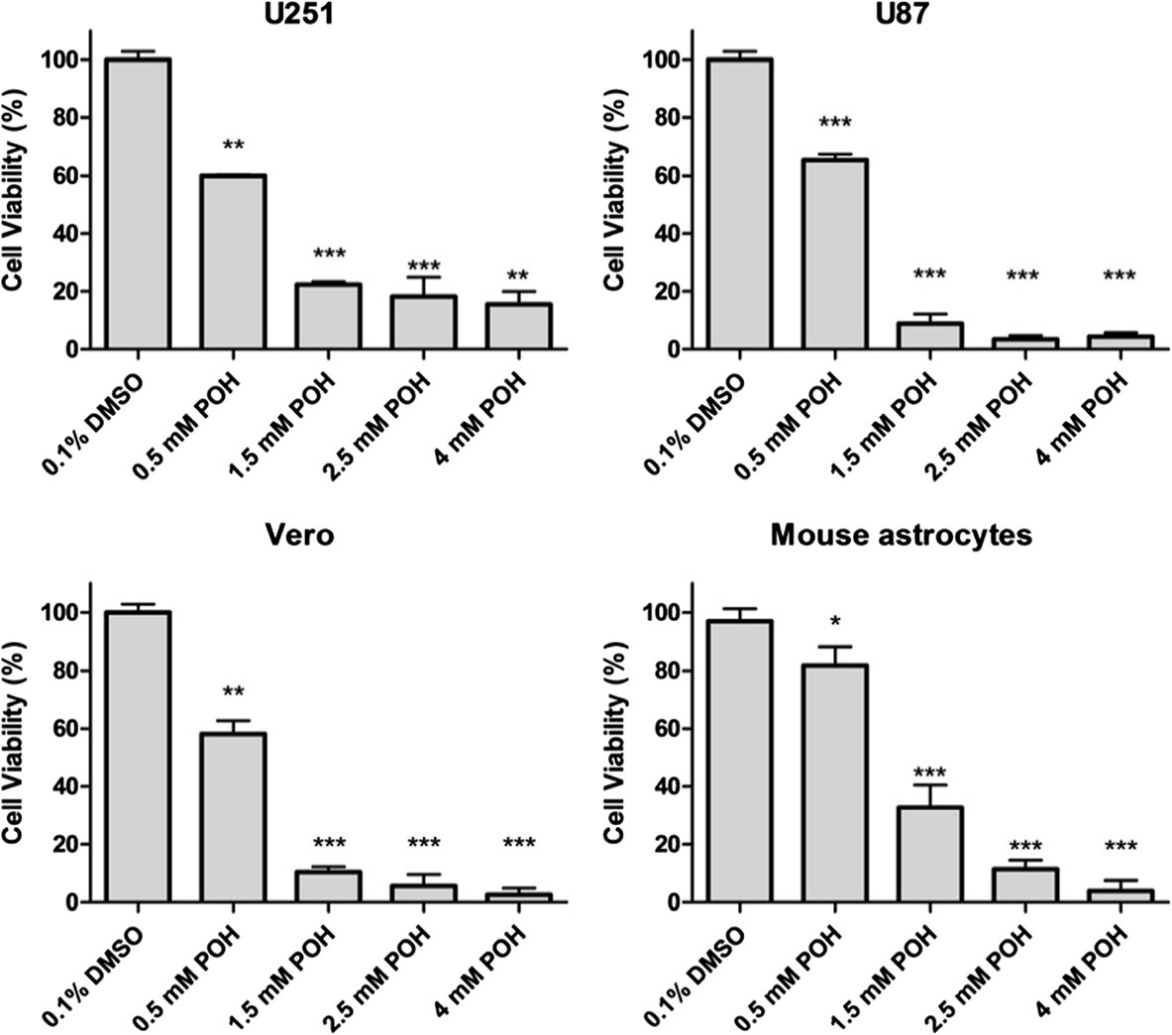
The effect of POH on cell viability. U251 and U87 cells, VERO cells and mouse astrocytes were treated with POH (0.5 - 4 mM) for 24 hours and the LDH activity was quantified. Each point represents the means ± SD from at least three different experiments. *p < 0.05, **p < 0.01, ***p < 0.001 vs. control group (0.1% DMSO), analyzed by Student’s t-test.

The cytotoxic effects of POH and PA in human GBM cells (U87 and U251) and non-tumor cells (VERO and mouse astrocytes) were measured. The cells were treated for 24 hours with different concentrations of POH (0.5, 1.5, 2.5 and 4 mM) and a high concentrations of PA (4 mM). Cell viability was measured using the LDH assay. The IC_50_ was 0.9 mM in VERO cells and 1.4 mM in mouse astrocytes. In U251 and U87 cells, the IC_50_ values were 1.4 and 1.1 mM, respectively (Figure 2). PA did not appreciably decrease the cell viabilities of any studied cells (Additional file 3).

### The effect of POH on JNK and p38 activation

**Figure 3:**
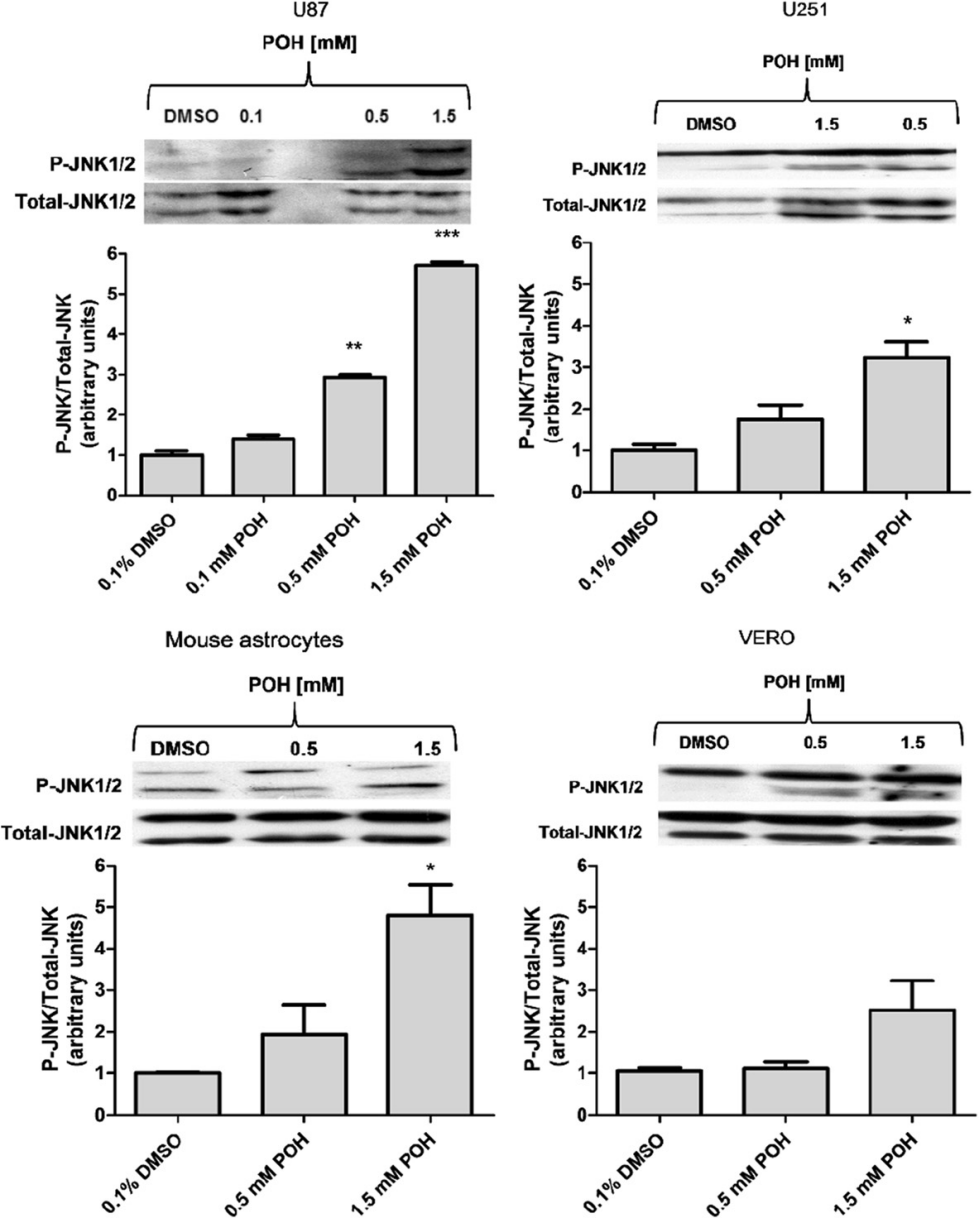
The effects of POH on the activation of JNK1/2 in U87 and U251 cells, VERO cells and mouse astrocytes. The cells were treated with POH for 30 minutes. The graph shows the densitometric analysis of p-JNK1/2 relative to the total JNK1/2 and is shown in arbitrary units as the ratio of the band densities from western blots of p-JNK1/2 and total JNK1/2 corrected for control. The figures are representative of three independent experiments. The graph represents the means ± SD from at least three different experiments. *p < 0.05, **p < 0.01, ***p < 0.001 vs. control group (0.1% DMSO), analyzed by Student’s t-test.

U87 cells were treated for 30 min with increasing doses of POH (0.1, 0.5 and 1.5 mM), which showed that POH increased JNK1/2 phosphorylation in a dose-dependent manner (Figure 3A). The same treatment was given to the U251 cell line, but the experiments were performed with 0.5 and 1.5 mM POH because 0.1 mM POH did not produce a significant effect in the U87 cell line. 1.5 mM POH significantly increased JNK1/2 phosphorylation in U251 cells (Figure 3B). Similar results to those found with U251 cells were obtained with mouse astrocytes, where 1.5 mM POH significantly increased JNK1/2 activation (Figure 3C). In VERO cells, POH did not significantly increase JNK1/2 activation (Figure 3D). The activation of p38 was assayed under the same conditions in the U87 cell line, and 1.5 mM POH significantly increased p38 phosphorylation (Additional file 4).

### The effects of methyl β-cyclodextrin and dasatinib on the activation of JNK by POH

**Figure 4:**
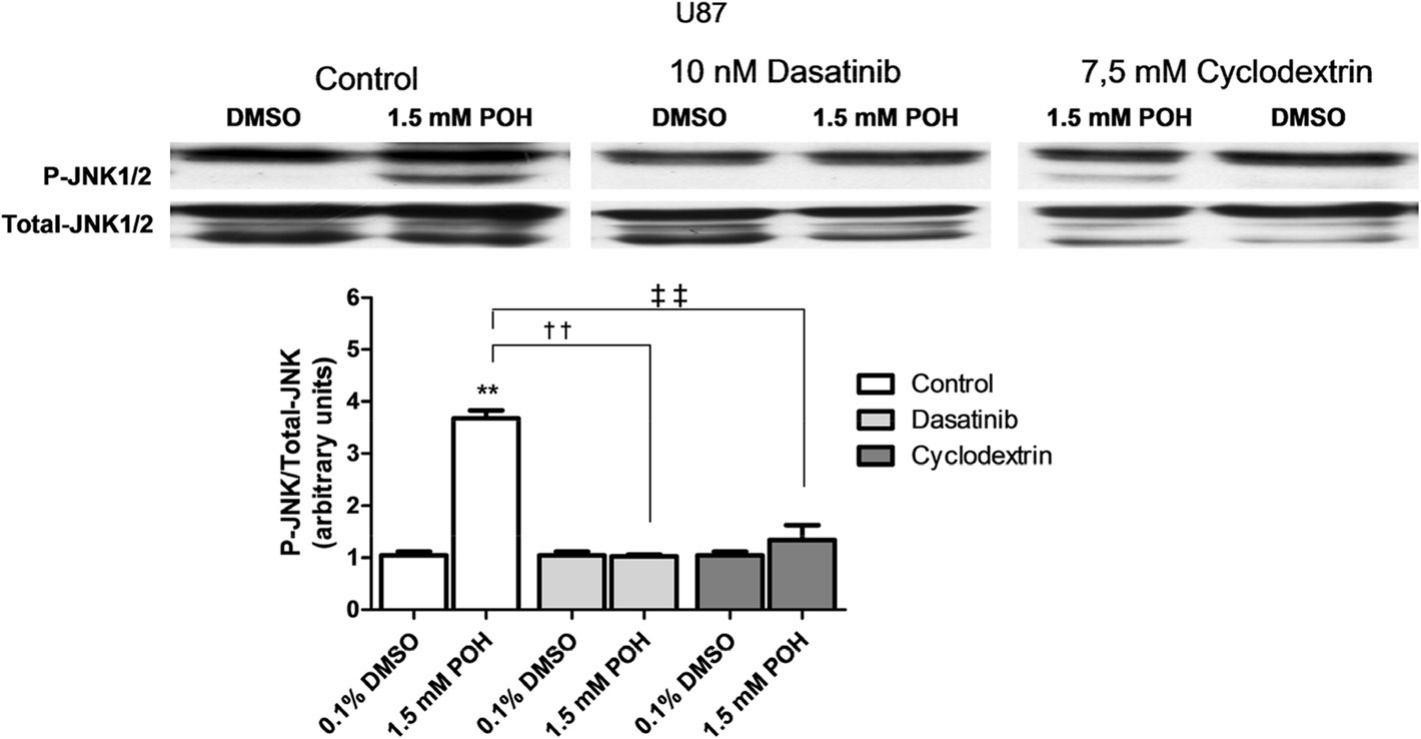
The effects of dasatinib and methyl β-cyclodextrin on the activation of JNK by POH in U87 cells. Cells were pretreated with 10 nM dasatinib and 7.5 mM methyl β-cyclodextrin for 30 minutes. After the pretreatment, DMSO (0.1%) or POH (1.5 mM) was added. After 30 minutes incubation, the expression of p-JNK1/2 and total JNK1/2 were detected by western blot. The data were expressed as the means ± SD from at least three different experiments. **p < 0.01 vs. control group (0.1% DMSO). ††p < 0.01, ‡‡p < 0.01 vs. control group (1.5 mM POH without pretreatment), analyzed by Student’s t-test.

U87 cells were pretreated for 30 minutes with 10 nM dasatinib and 7.5 mM methyl β-cyclodextrin. After this time, the cells were treated for an additional 30 minutes with 1.5 mM POH. The effects of POH on JNK1/2 activation were significantly reduced by both pretreatments (Figure 4).

### The effect of POH on cytokine release

**Figure 5:**
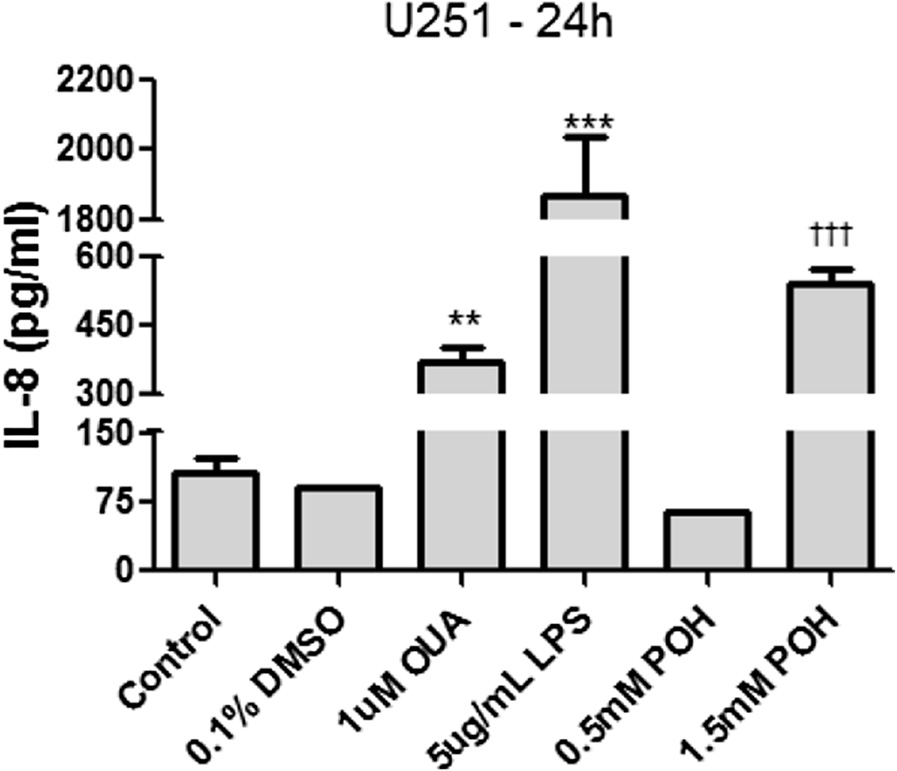
The effects of POH on the release of IL-8 in U251 cells. The cells were treated with 0.1% DMSO, POH (0.5 and 1.5 mM) or different positive controls (5 μg/mL LPS and 1 μM OUA) for 24 hours. An untreated control was also carried out. IL-8 detection was performed using an Enzyme-Linked Immunosorbent Assay (ELISA). The data were expressed as the means ± SD from at least three different experiments. **p < 0.01; ***p < 0.001 vs. control group (without treatment). ^†††^p < 0.001 vs. control group (0.1% DMSO), analyzed by One-way ANOVA followed by Newman-Keuls test.

During the three incubation periods used, POH did not alter the release of IL-1β, IL-6 and TNF-α in either of the two human GBM cell lines. Similar results were observed with IL-8 in both cell lines after 1 or 6 hours of incubation (the values were below the lowest detection limit of the ELISA kit used). On the other hand, an increase in the release of IL-8 after 24 hours of incubation with 1.5 mM POH was detected in the U251 cell line (Figure 5).

### The effect of JNK inhibition in the POH-mediated induction of cell death

**Figure 6:**
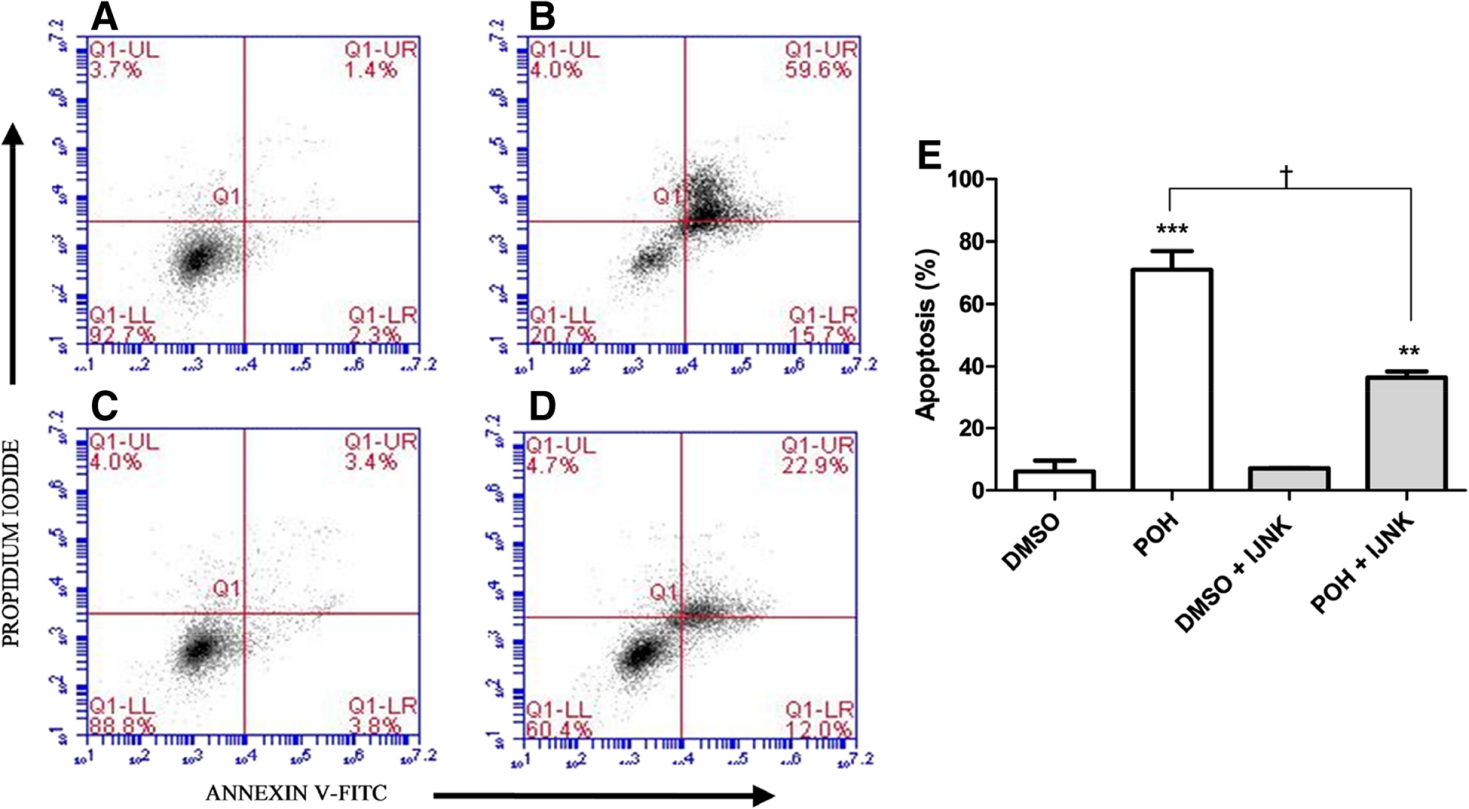
The effects of JNK inhibition on the induction of cell death by POH in U87 cells. Before treatment, U87 cells were incubated without **(A and B)** or with **(C and D)** JNK inhibitor V (0.5 μM) for 30 minutes. The cells were treated with 0.1% DMSO (A), 0.5 mM POH **(B)** 0.1% DMSO plus JNK inhibitor V **(C)**, and 0.5 mM POH plus JNK inhibitor V **(D)**. After 24 hours of incubation, the cells were stained with annexin V-FITC and propidium iodide and analyzed by flow cytometry. Figure 6E represents the percentage of dead cells indicated by early apoptosis and late apoptosis or necrosis (right lower quadrant + right upper quadrant, respectively), which was calculated from the data shown in Figures 6A-D. The data were expressed as the means ± SD from at least three different experiments. ***p < 0.001 vs. control group (0.1% DMSO), **p < 0.01 vs. control group (0.1% DMSO + JNK inhibitor V), †p < 0.05 vs. control group (0.5 mM POH without the JNK inhibitor V), analyzed by Student’s t-test.

**Figure 7:**
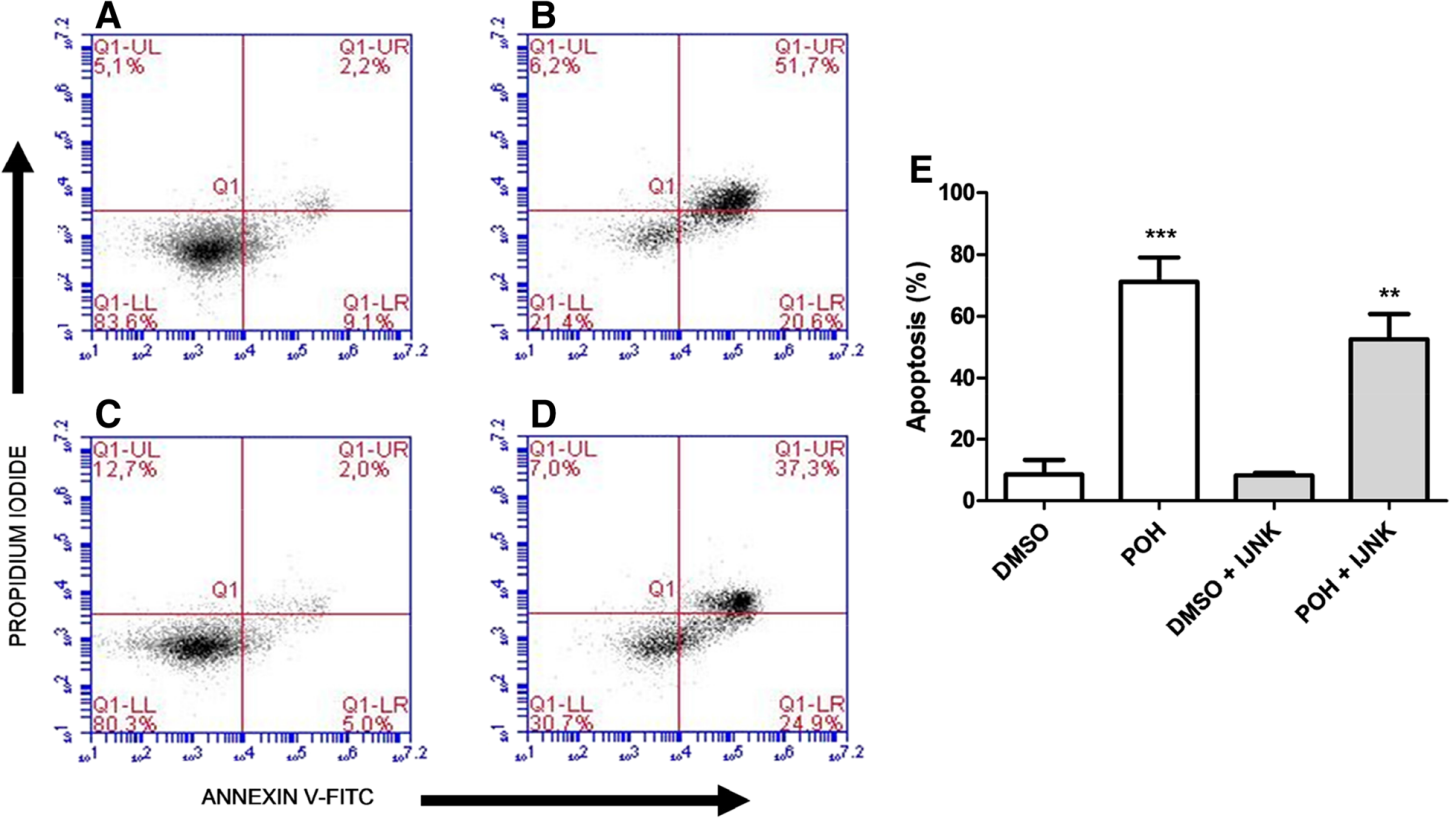
The effects of JNK inhibition on the induction of cell death by POH in U251 cells. Before treatment, U251 cells were incubated without **(A and B)** or with **(C and D)** JNK inhibitor V (0.5 μM) for 30 minutes. The cells were treated with 0.1% DMSO **(A)**, 0.5 mM POH **(B)** 0.1% DMSO plus JNK inhibitor V **(C)**, and 0.5 mM POH plus JNK inhibitor V **(D)**. After 24 hours of incubation, the cells were stained with annexin V-FITC and propidium iodide and analyzed by flow cytometry. Figure 7E represents the percentage of dead cells as indicated by early apoptosis and late apoptosis or necrosis (right lower quadrant + right upper quadrant, respectively), which was calculated from the data shown in Figures 7A-D. The data were expressed as the means ± SD from at least three different experiments. ***p < 0.001 vs. control group (0.1% DMSO), **p < 0.01 vs. control group (0.1% DMSO + JNK inhibitor V). The difference between the treated group (0.5 mM POH + JNK inhibitor V) and the control group (0.5 mM POH without the JNK inhibitor V) was not statistically significant. The data were analyzed by Student’s t-test.

U87 and U251 cells were pretreated for 30 min with 0.5 μM JNK inhibitor V. Controls without the pretreatment were also conducted. Then, the treatment was carried out with the addition of 0.1% DMSO or 0.5 mM POH either with or without 0.5 μM JNK inhibitor V for 24 hours. Cell death was determined by flow cytometry. Propidium iodide and annexin V-FITC fluorescence analysis revealed populations of viable and dead cells, as shown in the respective quadrants (Figure 6 and 7). The lower left quadrant shows the viable cells. The lower right and upper right quadrant represent early apoptosis and late apoptosis or necrosis, respectively. The upper left quadrant represents necrosis.

U87 cells in the control group were treated only with 0.1% DMSO and are shown in Figure 6A. Treatment with 0.5 mM POH (Figure 6B) showed a significant increase in the dead cell population (71% ± 6.08) compared to the control group. In addition, cell groups that were pretreated with JNK inhibitor V (0.5 μM) and post-treated with the same inhibitor plus 0.1% DMSO (Figure 6C) or 0.5 mM POH (Figure 6D) were evaluated. In this case, 36.4% (±2.05) of the cells died when treated with POH plus JNK inhibitor V (Figure 6D). This value was higher than the control group (Figure 6C), but significantly lower than the rate of cell death in the POH-treated group (Figure 6B). These results are shown in Figure 6E.

Using the U251 cell line, 0.5 mM POH also significantly increased the cell death (71.2% ± 7.9) (Figure 7B) compared to the control group (0.1% DMSO) (Figure 7A). Cells treated with POH plus JNK inhibitor V showed approximately 52.6% (±8.02) cell death (Figure 7D). This value was meaningful in comparison with the control group (Figure 7C) but insignificant when compared to the POH-treated group (Figure 7B). These results are presented in Figure 7E.

### Induction of apoptosis by POH

U87 and U251 cells were treated with 0.5 mM POH and 0.5 mM POH plus 0.5 μM JNK inhibitor V for 24 hours. The treatment was also carried out with the addition of DMSO (0.1%) or JNK inhibitor V. Before treatment, some groups of cells were pretreated with JNK inhibitor V (0.5 μM) for 30 minutes. Cell death by apoptosis was determined by immunocytochemistry.

**Figure 8:**
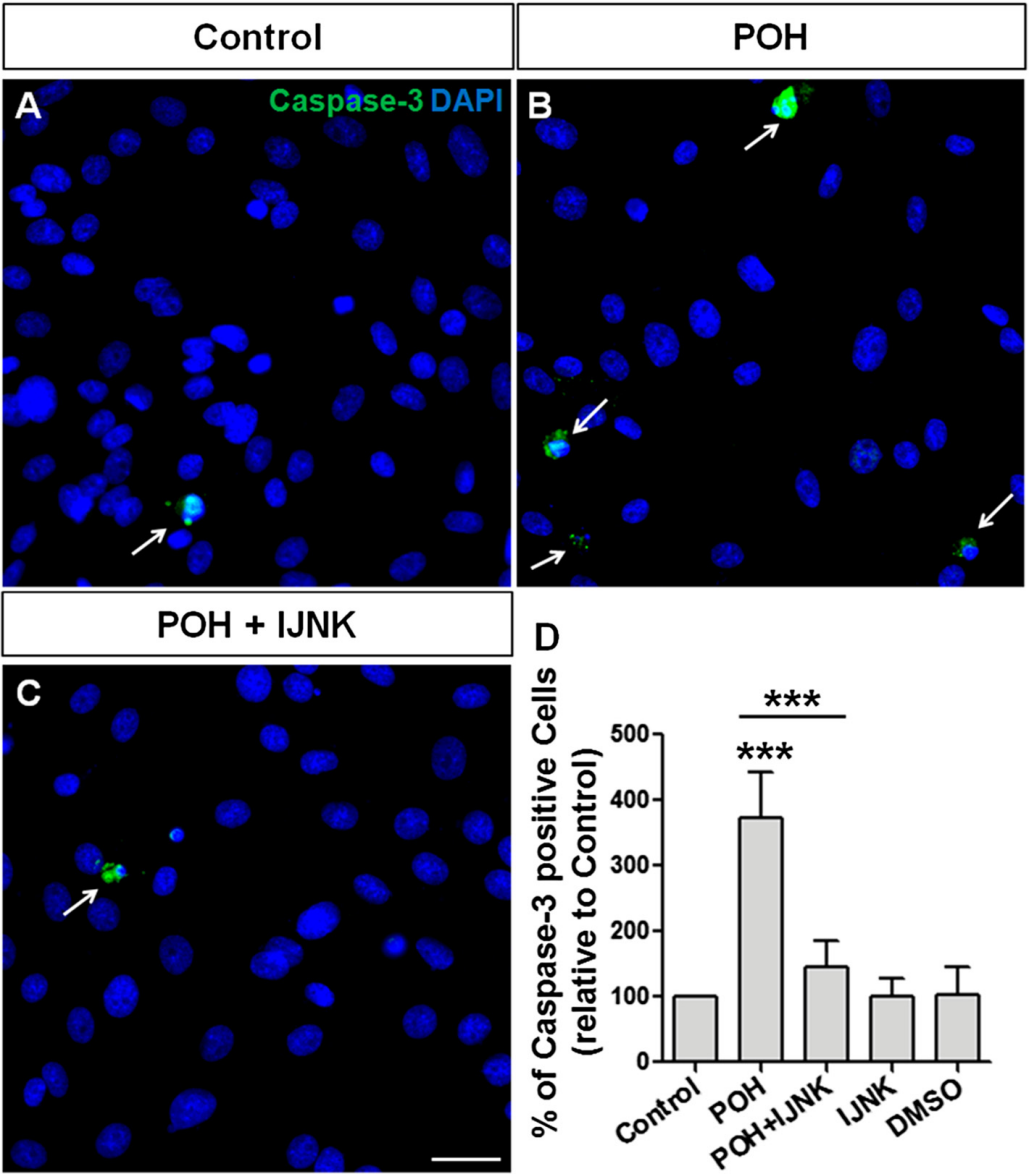
POH-induced apoptosis in U87 cells. U87 in the control condition (Control, **A)**. The cells were treated with 0.5 mM POH (POH, **B)** and 0.5 mM POH plus 0.5 μM JNK inhibitor V (POH + IJNK, **C)**. After 24 hours, the cells were immunostained for cleaved caspase-3 and the number of positive cells was analyzed **(D)**. Whereas POH induced cell apoptosis, the addition of the JNK inhibitor completely inhibited this effect. The addition of DMSO or JNK inhibitor V alone had no effect on cell death. Scale bar: 20 μm.*P < 0.050, analyzed by Student’s t-test.

**Figure 9:**
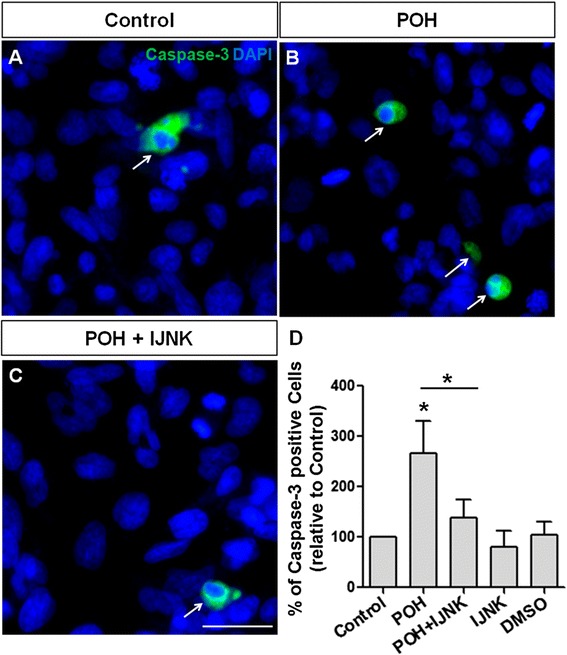
POH-induced apoptosis in U251 cells. U251 in the control condition (Control, **A)**. The cells were treated with 0.5 mM POH (POH, **B)** and 0.5 mM POH plus 0.5 μM JNK inhibitor V (POH + IJNK, **C)**. After 24 hours, the cells were immunostained for cleaved caspase-3 and the number of positive cells was analyzed **(D)**. Whereas POH induced cell apoptosis, the addition of the JNK inhibitor completely inhibited this effect. The addition of DMSO or JNK inhibitor V alone had no effect on cell death. Scale bar: 20 μm.*P < 0.050, analyzed by Student’s t-test.

POH-induced apoptosis in U87 and U215 cells are shown Figure 8B and 9B, respectively. The cells were immunostained for cleaved caspase-3 and the number of positive cells was analyzed (8D and 9D). JNK1/2 inhibition substantially reduced the amount of POH-induced apoptosis (Figure 8C and 9C). The control conditions are shown in Figures 8A and 9A. The addition of JNK inhibitor V alone had no effect on cell death.

## Discussion

Perillyl alcohol (POH) has the ability to induce apoptosis in cancer cells [37-40] and has been used in the therapy of different tumors, including gliomas [31,32,41,42]. These effects of POH may be at least partly related to the inhibition of NKA activity [28,43].

As a first step in studying the ability of POH to inhibit glioma cell proliferation, we evaluated the NKA activity based on the incorporation of Rb^+^ by tumor and non-tumor cells. POH inhibited the NKA activity in a dose-dependent manner. The IC_50_ values of U251 and U87 human GBM cell lines were similar and were very close to the value previously found by our group using the A172 human GBM cell line [28]. In these three human GBM cell lines (U251, U87 and A172), POH completely inhibited Rb^+^ uptake at 4 mM. In the non-tumor VERO cell line, the IC_50_ was slightly higher and POH did not reach 100% inhibition at 4 mM. These IC_50_ values were not significantly different, which was expected because kidney and GBM cells express the same α1 NKA isoform [23,44], against which POH has a higher degree of selectivity [28]. Due to the unavailability of normal human astrocyte cultures, we used mouse astrocytes. In these cells, 2 mM POH inhibited completely the Rb^+^ uptake. It is interesting to note that mouse astrocytes mainly express the NKA α2 and α3 isoforms, which are more sensitive to the inhibitory effects of POH [28,44]. Furthermore, perillic acid (PA), a metabolite produced rapidly in the human body after POH administration, was also tested. NKA activity was not affected by PA in any of the cell lines used in this work. To assess whether the inhibition of Rb^+^ uptake by cells (U251, U87, VERO and mouse astrocytes) were caused by cell death, cell viability experiments were carried out in the presence of POH, PA and ouabain (OUA). The cell viability was significantly decreased by POH only when a high concentration was used. OUA and PA did not affect the cell viability. Therefore, the decreased Rb^+^ uptake occurred through NKA inhibition and not due to cell death.

Low plasma levels of POH cannot be measured accurately, but the levels of their metabolites are detectable due to the rapid degradation of POH [45]. Thus, POH administration is more advantageous than the use of cardiac glycosides because its rapid metabolism decreases its undesirable adverse effects. Interestingly, therapeutic doses of POH are far superior to those of cardiac glycosides [46]. The cytotoxic properties of compounds that may or may not be similar to cardiac glycosides but can still affect NKA activity have already been tested [47-52]. Though PA did not affect the cell viability of the studied cells, POH had a potential cytotoxic effect on all cell lines. The cell viabilities of both tumor and non-tumor cells were significantly reduced at lower dose and increased thereafter according to the POH dose. The IC_50_ value for POH was not significantly different between cell types (U251 and U87). Similar results were obtained by Cho *et al.* (2012) [53] using glioma cell lines U87, U251 and LN229.

Due to its role in fundamental cellular functions such as proliferation, differentiation and apoptosis, NKA has been considered as a target for drugs, especially those with antitumor activities [17,25,52]. Several studies have reported the induction of apoptosis by cardiac glycosides in various tumor cells [14,24,46,54-56]. At this point, it is important to emphasize that POH induces apoptosis in various tumor cells, including gliomas. However, the exact mechanism by which this drug induces apoptosis is unclear [38,57]. It is known that POH preferentially inhibits the NKA α1 isoform [28], which modulates apoptosis, cell migration and proliferation and is overexpressed in the caveolae of GBM cells [26,27]. Considering these facts, we tested the activation of JNK, a main protein target of the MAPK pathway controlling cell growth and/or death [15].

The human GBM cells (U87 and U251) and non-tumor cells (VERO cells and mouse astrocytes) were treated with POH. POH increased JNK1/2 phosphorylation in the U87 and U251 cell lines that was similar to mouse astrocytes but not VERO cells. Studies with fibroblasts have shown that JNK activation and the pro-apoptotic protein Bax, a Bcl2 family member [58], are sufficient to cause the caspase-independent release of cytochrome *c*, which leads to apoptosis. Additionally, POH increases the Bax expression in non-small cell lung cancers [39,40]. Although JNK phosphorylation by POH has not yet been described, Satomi *et al*. (1999) [59] showed that POH induces the increased expression and phosphorylation of c-Jun protein in breast cancer cells, which is involved in cellular proliferation and apoptosis. This phosphorylation occurred quickly at the N-terminal site of c-Jun, which is normally phosphorylated by JNK. According to these researchers, c-Jun activation by POH in these cells may represent a relevant early response to apoptosis. Thus, POH seems to modulate the JNK signaling cascade [59]. In addition, the activation of p38, another protein of the MAPK family, was observed in U87 cells, broadening the framework of intracellular signaling proteins involved in POH-induced cell death. With respect to JNK and p38 activation in GBM cells, it was recently reported that piperlongumine, an alkaloid with lipophilic properties found in plants of the species *Piper longum L*., can activate JNK and p-38 MAPKs, leading to apoptosis of U87, LN229 and 8MG-BA GBM cell lines through the accumulation of reactive oxygen species [60].

To correlate the POH induced-JNK phosphorylation with NKA, we evaluated JNK activation in the presence of the Src kinase inhibitor dasatinib [61] and methyl β-cyclodextrin, a molecule that extracts cholesterol from the plasma membrane, disrupting lipid rafts [62] and thus blocking the MAPK pathway in the signalosome, which is a caveolae microdomain in which the formation of the NKA-Src complex occurs. NKA mediated-cellular signaling is initialized in the cholesterol and sphingomyelin-rich plasma membrane subfractions of the caveolae, in which the NKA α1 subunit interacts with various signaling proteins. Src is a primary target of the NKA α1 subunit and is responsible for communication between NKA and other proteins. When the NKA-Src complex is activated, different signaling pathways, including the MAPK pathways, are initiated in a specific manner depending on the stimulus and cell type [7,63]. In U87 cells, the effect of POH on JNK1/2 activation was significantly reduced by dasatinib and methyl β-cyclodextrin pretreatment. Both pretreatments appeared to prevent NKA activation in the signalosome. These results support our hypothesis that NKA is directly involved in mechanisms involving POH-mediated JNK activation in human GBM cells, triggering cell death through the activation of NKA in the signalosome.

Activated MAPKs are able to mediate the release of interleukin (IL) [64] and can be induced by cardiac glycosides through NKA in cytotrophoblast embryonic cells [15]. However, POH suppressed the level of pro-inflammatory cytokines in ischemia-reperfusion injury in rat brains [65]. In GBM, the presence of pro-inflammatory cytokines is associated with tumor growth and hence with its malignancy. IL-6 induces the proliferation of tumor cells proliferation, whereas IL-8 has angiogenic and chemotactic properties [66]. During the three incubation periods used (1, 6 and 24 hours), POH did not alter the release of pro-inflammatory cytokines in either of the two human GBM cell lines. On the other hand, an increase in the release of IL-8 after 24 hours of incubation with POH was detected in the U251 cell line. Cho *et al*. (2012) [53] found a decrease in the release of IL-8 in U87 cells after 48 hours of incubation using 0.6 mM POH. Since IL-8 enhances GBM invasion [67,68], this increase may be a strategy used by the cells in circumventing the POH-induced effects on cell death.

POH showed a significant increase in early and late apoptosis or necrosis of U87 and U251 cells, but when these cells were pretreated with a JNK inhibitor, a significant decrease in cell death was found. Our results in the U251 and U87 human GBM cell lines showed that the inhibition of JNK activation decreased the effects caused by POH, demonstrating the possible involvement of JNK in the induction of apoptosis in GBM cells. However, the reduction in the POH-induced cell death did not occur to the same extent in both cell lines. Interestingly, in addition to this difference in the rates of blocked cell death between these tumor cell lines, the effect of POH on the activation of JNK was also lower in the U251 cells, as previously shown. Since the same intensity of cell death was induced by POH in both GBM cell lines, part of this pathway might be JNK-independent in U251 cells. It is possible that the activation of other MAPKs or the involvement of the PI3K/Akt pathway are also linked to the NKA-Src complex [62,68].

Human GBM cells are driven to undergo apoptosis when treated with POH [38,57]. As shown previously, JNK activation appears to be involved in this phenomenon. To confirm the involvement of apoptosis in POH-mediated cell death, U87 and U251 cells were treated with POH or POH plus JNK inhibitor V and then were immunostained for cleaved caspase-3. As expected, POH induced apoptosis in GBM cells, though interestingly, JNK inhibition decreased the POH-induced apoptosis. NKA, especially the α1 isoform, plays an important role in GBM survival [26,27]. Inhibitors of this enzyme, particularly the cardiac glycoside UNBS1450 and the monoterpene POH, cause death in these tumor cells by autophagy and apoptosis induction, respectively [26,38]. However, the mechanisms triggering apoptosis were not completely elucidated [38].

## Conclusions

**Figure 10:**
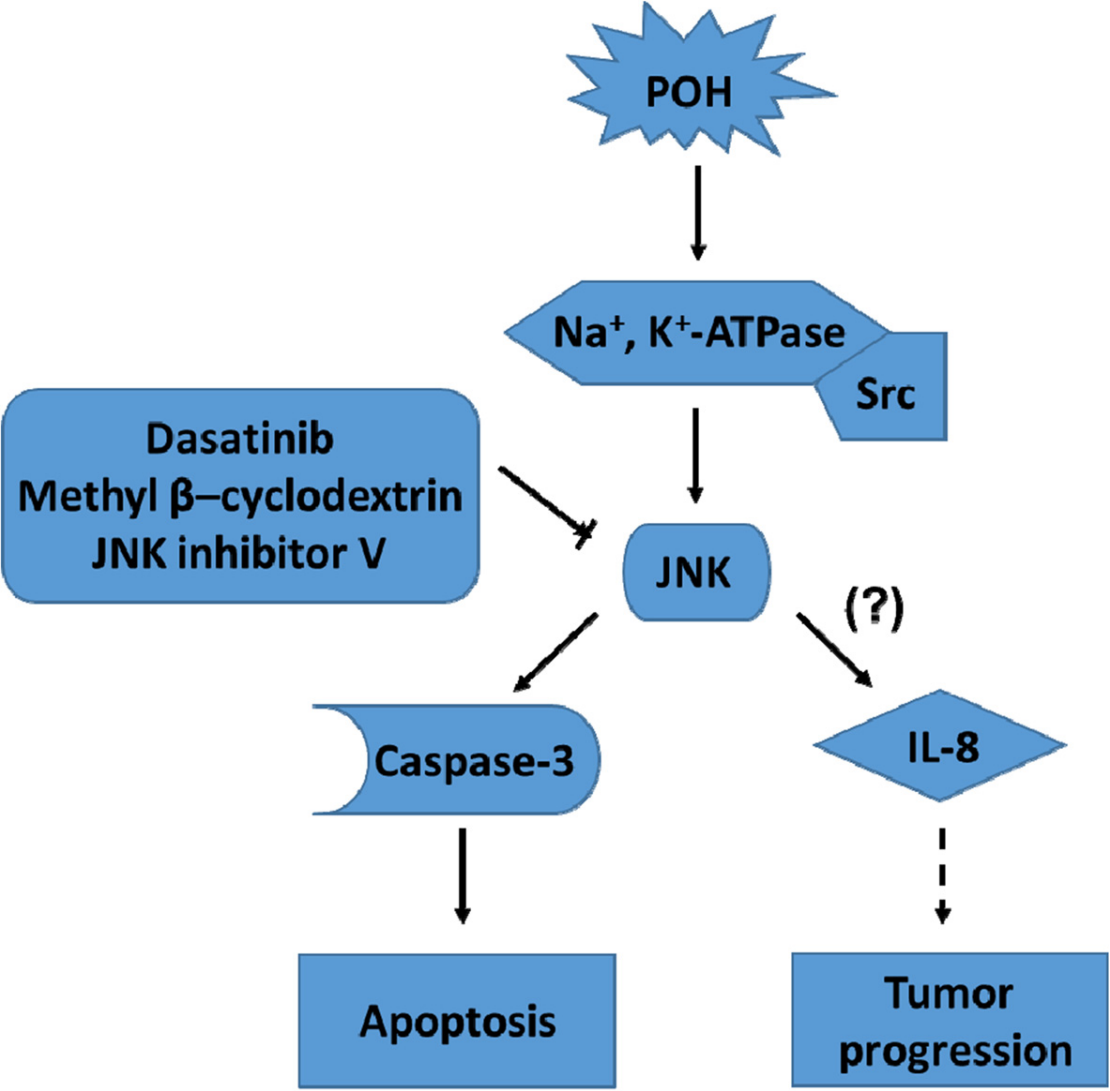
A schematic diagram showing the possible relationship between apoptosis in Glioblastoma cells and the NKA-Src-JNK pathway. The connection of POH to the NKA-Src complex in the signalosome leads to the activation of JNK. Src inhibition (by dasatinib) and caveolae disruption (by methyl β-cyclodextrin) blocked the phosphorylation of JNK. JNK activation can induce apoptosis through cleavage of caspase-3. JNK inhibition (by the JNK inhibitor V) decreased the induction of apoptosis. The JNK-mediated production of IL-8 in GBM cells has not been described in the literature but an increase in the release of IL-8 might favor tumor progression. The solid lines indicate the findings of this study, whereas the dashed line show the known molecular mechanisms. POH: Perillyl alcohol; NKA: Na/K-ATPase; Src: Src Kinase; JNK: c-Jun N-terminal Kinase; and IL-8: Interleukin 8.

Together, our data lead to the following proposed mechanism for the action of POH in tumor glial cells (Figure 10): the connection between POH and NKA present in the signalosome could stimulate the Src kinase, leading to signaling via the MAPK signaling cascade and subsequent activation of JNK and p38. Activated JNK could induce apoptosis through different molecular mechanisms, including caspase-3 activation. Although JNK-mediated IL-8 production in GBM cells has not been described in the literature, we observed an increase in the release of this cytokine in U251 cells, which may favor tumor progression. This hypothesis is supported by our observation that 1) Src inhibition and caveolae disruption blocked the phosphorylation of JNK, and 2) the induction of apoptosis was reduced by JNK inhibition. Here, we demonstrate that the caveolar NKA-Src complex signals through JNK and caspase-3 activation during the POH-induced apoptosis of human GBM cells.

## Additional file

Additional file 1:
**The effect of PA on the activity of NKA in the U251 and U87 cell lines, VERO cells and mouse astrocytes.** Cells were treated with 4mM PA for 30 minutes. The NKA activity was expressed as the difference between the Rb^+^ uptake in the absence or presence of 0.5 mM OUA. Each point represents the means ± SD from at least four different experiments conducted in triplicate. Additional file 2:
**The effect of POH, PA and OUA on cell viability.** U251 and U87 cells, VERO cells and mouse astrocytes were treated with POH (0.5 - 4 mM), 4mM PA and 0.5mM OUA for 30 minutes and the LDH activity was quantified. Each point represents the means ± SD from at least three different experiments. ***p<0.001 vs. control group (0.1% DMSO), analyzed by Student’s t-test. Additional file 3:
**The effect of PA on cell viability.** U251 and U87 cells, VERO cells and mouse astrocytes were treated with 4mM PA for 24 hours and the LDH activity was quantified. Each point represents the means ± SD from at least three different experiments. Additional file 4:
**The effects of POH on the activation of p38 in U87 cells.** The cells were treated with POH for 30 minutes. The graph shows the densitometric analysis of p-p38 relative to the total p38 and is shown in arbitrary units as the ratio of the band densities from western blots of p-p38 and total p38 corrected for control. The figures are representative of three independent experiments. The graph represents the means ± SD from at least three different experiments. **p<0.01 vs. control group (0.1% DMSO), analyzed by Student’s t-test.
