# Supplementary material for: Na/K-ATPase as a target for anticancer drugs: studies with perillyl alcohol
Source: Mol Cancer. 2015 May 15;14:105. doi: 10.1186/s12943-015-0374-5 (PMC4432499; doi:10.1186/s12943-015-0374-5)
Supplement: Additional file 1: — The effect of PA on the activity of NKA in the U251 and U87 cell lines, VERO cells and mouse astrocytes. Cells were treated with 4mM PA for 30 minutes. The NKA activity was expressed as the difference between the Rb+ uptake in the absence or presence of 0.5 mM OUA. Each point represents the means ± SD from at least four different experiments conducted in triplicate. [file 12943_2015_374_MOESM1_ESM.doc]

Additional file 1


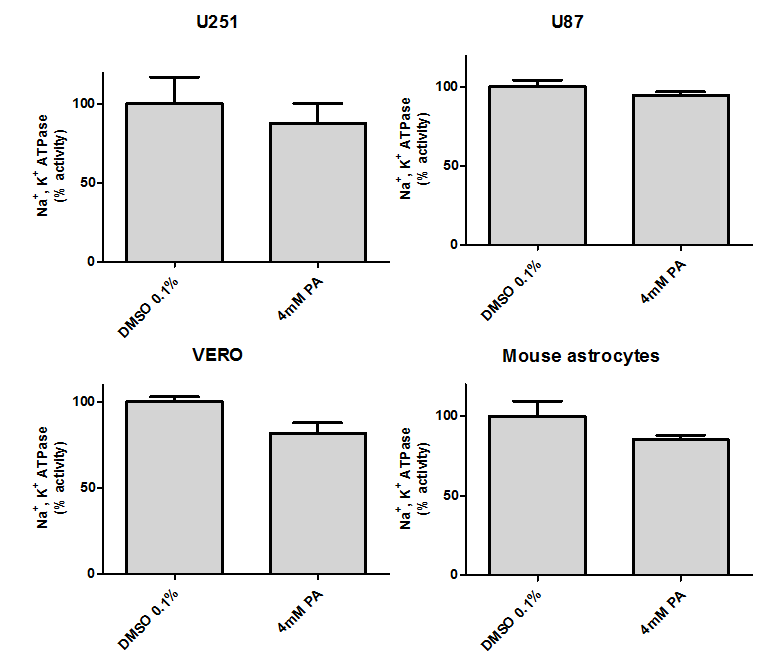


**Additional file 1:** The effect of PA on the activity of NKA in the U251 and U87 cell lines, VERO cells and mouse astrocytes. Cells were treated with 4mM PA for 30 minutes. The NKA activity was expressed as the difference between the Rb^+^ uptake in the absence or presence of 0.5 mM OUA. Each point represents the means ± SD from at least four different experiments conducted in triplicate.
