# Supplementary material for: Na/K-ATPase as a target for anticancer drugs: studies with perillyl alcohol
Source: Mol Cancer. 2015 May 15;14:105. doi: 10.1186/s12943-015-0374-5 (PMC4432499; doi:10.1186/s12943-015-0374-5)
Supplement: Additional file 2: — The effect of POH, PA and OUA on cell viability. U251 and U87 cells, VERO cells and mouse astrocytes were treated with POH (0.5 - 4 mM), 4mM PA and 0.5mM OUA for 30 minutes and the LDH activity was quantified. Each point represents the means ± SD from at least three different experiments. ***p<0.001 vs. control group (0.1% DMSO), analyzed by Student’s t-test. [file 12943_2015_374_MOESM2_ESM.doc]

Additional file 2


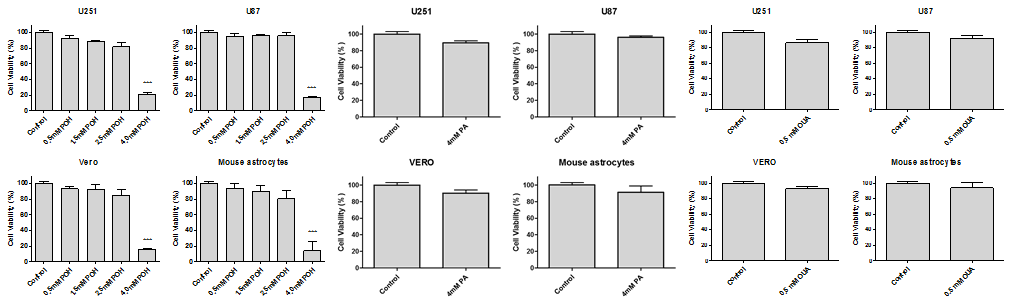


**Additional file 2:** The effect of POH, PA and OUA on cell viability. U251 and U87 cells, VERO cells and mouse astrocytes were treated with POH (0.5 - 4 mM), 4mM PA and 0.5mM OUA for 30 minutes and the LDH activity was quantified. Each point represents the means ± SD from at least three different experiments. *** p<0.001 vs. control group (0.1% DMSO), analyzed by Student’s t-test.
