# Supplementary material for: Na/K-ATPase as a target for anticancer drugs: studies with perillyl alcohol
Source: Mol Cancer. 2015 May 15;14:105. doi: 10.1186/s12943-015-0374-5 (PMC4432499; doi:10.1186/s12943-015-0374-5)
Supplement: Additional file 3: — The effect of PA on cell viability. U251 and U87 cells, VERO cells and mouse astrocytes were treated with 4mM PA for 24 hours and the LDH activity was quantified. Each point represents the means ± SD from at least three different experiments. [file 12943_2015_374_MOESM3_ESM.doc]

Additional file 3


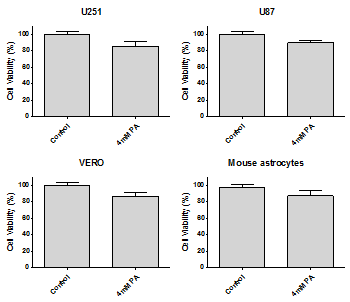


**Additional file 3:** The effect of PA on cell viability. U251 and U87 cells, VERO cells and mouse astrocytes were treated with 4mM PA for 24 hours and the LDH activity was quantified. Each point represents the means ± SD from at least three different experiments.
