# Supplementary material for: Na/K-ATPase as a target for anticancer drugs: studies with perillyl alcohol
Source: Mol Cancer. 2015 May 15;14:105. doi: 10.1186/s12943-015-0374-5 (PMC4432499; doi:10.1186/s12943-015-0374-5)
Supplement: Additional file 4: — The effects of POH on the activation of p38 in U87 cells. The cells were treated with POH for 30 minutes. The graph shows the densitometric analysis of p-p38 relative to the total p38 and is shown in arbitrary units as the ratio of the band densities from western blots of p-p38 and total p38 corrected for control. The figures are representative of three independent experiments. The graph represents the means ± SD from at least three different experiments. **p<0.01 vs. control group (0.1% DMSO), analyzed by Student’s t-test. [file 12943_2015_374_MOESM4_ESM.doc]

Additional file 4


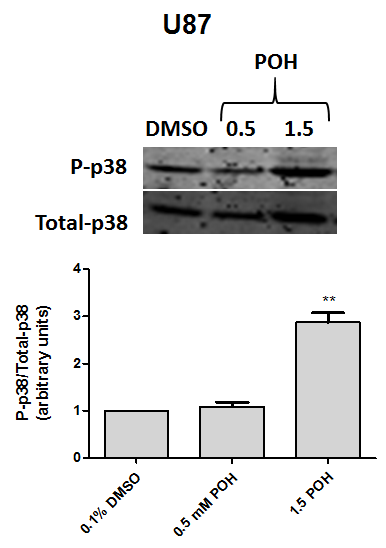


**Additional file 4:** The effects of POH on the activation of p38 in U87 cells. The cells were treated with POH for 30 minutes. The graph shows the densitometric analysis of p-p38 relative to the total p38 and is shown in arbitrary units as the ratio of the band densities from western blots of p-p38 and total p38 corrected for control. The figures are representative of three independent experiments. The graph represents the means ± SD from at least three different experiments. ** p<0.01 vs. control group (0.1% DMSO), analyzed by Student’s t-test.
